# Identification of Predictive Factors for Lymph Node Metastasis in pT1 Stage Colorectal Cancer Patients: A Retrospective Analysis Based on the Population Database

**DOI:** 10.3389/pore.2022.1610191

**Published:** 2022-02-28

**Authors:** Jiawei Song, Huanhuan Yin, Yong Zhu, Shengqi Fei

**Affiliations:** ^1^ Department of Gastrointestinal Surgery, Changxing People’s Hospital, Changxing, China; ^2^ Department of Gastroenterology, Changxing People’s Hospital, Changxing, China

**Keywords:** colorectal cancer, lymph node metastasis, SEER, predictive factors, pT1 stage

## Abstract

**Objective:** The purpose of this study was to identify predictive factors for lymph node metastasis (LNM) in pT1 stage colorectal cancer (CRC) patients.

**Methods:** From the Surveillance, Epidemiology, and End Results (SEER) database, 2,697 consecutive pT1 stage patients who underwent surgical resection were retrospectively reviewed. Predictive factors for LNM were identified by the univariate and multivariate logistic regression analysis. The Kaplan-Meier curves and multivariate Cox regression analysis were used to evaluate the relationships between LNM and overall survival (OS) as well as cancer specific survival (CSS) of pT1 stage CRC patients.

**Results:** The prevalence of LNM in pT1 stage CRC patients was 15.2% (410/2,697). Patient age <60 years (OR:1.869, 95% CI: 1.505–2.321, *p* < 0.001), poorly differentiated or mucinous or signet ring cell adenocarcinoma (OR:2.075, 95% CI: 1.584–2.717, *p* < 0.001), elevated carcinoembryonic antigen (CEA) level (OR:1.343, 95% CI: 1.022–1.763, *p* = 0.033) and perineural invasion (PNI) (OR:6.212, 95% CI: 3.502–11.017, *p* < 0.001) were significantly associated with LNM in pT1 stage patients. The survival analysis demonstrated that pT1 stage patients with LNM had a worse OS (5-year OS: 82.2% vs 88.7%, *p* = 0.020) and CSS (5-year CSS: 74.9% vs 81.5%, *p* = 0.041) than those without lymph node metastasis. Lymph node metastasis was an independent predictor of poor OS (HR: 1.543, 95% CI: 1.156–2.060, *p* = 0.003) and CSS (HR: 1.614, 95% CI: 1.121–2.324, *p* = 0.010) for pT1 stage colorectal cancer patients.

**Conclusion:** Age, differentiation type, CEA level and perineural invasion were independent predictive factors for LNM in pT1 stage CRC patients. These findings might provide further risk stratification for pT1 stage patients and help clinicians identify high-risk individuals.

## Introduction

Colorectal cancer (CRC) is one of the most prevalent malignant tumors and a major cause of cancer-related mortality in both Eastern and Western populations [1,2]. With the advancement in endoscopic techniques and screening programs, an increasing number of colorectal cancer patients were diagnosed at a relatively early stage. Although endoscopic resection has become an alternative treatment method for submucosal invasive (pT1 stage) colorectal cancer patients [3,4], the potential risk of lymph node metastasis is still an important clinical consideration. It has been demonstrated that lymphovascular invasion, poorly differentiated adenocarcinoma or mucinous or signet-ring cell carcinoma, deep submucosal invasion (≥1,000 μm) and tumor budding were independent predictive factors for lymph node metastasis in pT1 stage colorectal cancer patients [5–8]. According to current treatment guidelines [9,10], patients with any of these risk factors were recommended to receive additional surgical treatment with lymph node dissection after endoscopic resection. However, some studies reported that the incidence of lymph node metastasis in patients with a single risk factor such as submucosal invasion ≥1,000 μm was only 1.6%–2.2%, which was extremely low in comparison to the overall prevalence of lymph node metastasis in pT1 patients [6,11]. To reduce the possibility of additional surgery, the accurate prediction of lymph node metastasis is crucial to determine the candidate for endoscopic resection. On the other hand, the frequency of lymph node metastasis was approximately 6.8%–14.0% even after additional surgery [12,13]. Therefore, additional surgery may be not always necessary for patients who underwent potential non-curative endoscopic resection. Further risk assessment for lymph node metastasis in pT1 stage colorectal cancer patients was warranted to determine the suitable treatment strategy.

In the present study, we analyzed the specific clinicopathological features and identified predictive factors for lymph node metastasis in pT1 stage colorectal cancer patients using the Surveillance, Epidemiology, and End Results (SEER) database.

## Materials and Methods

### Patients Selection and Data Collection

From the SEER 18 Registries Research database (1973–2016), the data on consecutive patients who underwent surgical resection for pT1 stage colorectal cancer between January 2010 and December 2015 were retrospectively identified and analyzed. This study was further restricted to patients with primary colorectal adenocarcinoma or mucinous adenocarcinoma or signet ring cell (SRC) carcinoma confirmed by histopathology. The patients were excluded from this study if they had distant metastasis at presentation or received local excision. Moreover, we excluded those patients with missing data on relevant clinicopathologic variables. Also, the patients aged 18 years or younger were not included in the study. According to the inclusion and exclusion criteria, a total of 2,697 consecutive colorectal cancer patients were eligible for this analysis.

Demographic and clinicopathologic data, including patient age, sex, race, year of diagnosis, tumor location, preoperative carcinoembryonic antigen (CEA) level, pathological T category (pT stage), pathological N category (pN stage), pathological TNM stage, tumor size, histological classification, differentiation type, perineural invasion (PNI) and lymph node yield, were collected and analyzed. The protocol of this study was approved by the Ethics Committee of Changxing People’s Hospital. The informed consent was exempted because the information of all patients was de-identified and available from a public database.

### Statistical Analysis

Categorical variables were reported as absolute numbers and percentages, and continuous variables were expressed as mean with standard deviation (SD) if normal distribution data; otherwise, they were reported as the median and its interquartile range (IQR). The differences of clinicopathologic features between patients with and without lymph node metastasis were compared using Pearson’s chi-square test or Fisher’s exact for categorical variables. The predictive factors of lymph node metastasis for pT1 stage colorectal cancer patients were evaluated by the univariate and multivariate Logistic regression analysis, and the data were estimated as odd ratio (OR) and 95% confidence interval (CI). The Kaplan-Meier curves were plotted to evaluate the effects of lymph node metastasis on the overall survival (OS) and cancer specific survival (CSS) of pT1 stage colorectal cancer patients. The multivariate Cox regression analysis was used to further determine the independent prognostic significance of lymph node metastasis for these patients. All data processing and statistical analysis were carried out using R software program with version 3.6.1 (http://www.r-project.org), and the statistical significance was accepted at a *p*-value < 0.05.

## Results

### Patients Characteristics of Study Cohort

The entire cohort consisted of 1,444 males (53.5%) and 1,253 females (46.5%), and the proportion of patients aged 60 or older was 59.0% (*n* = 1,592). The median of tumor size was 2.0 cm (IQR: 1.3–2.8), and 463 of 2,697 patients (17.2%) had an elevated CEA level before surgery. Of patients, 1,984 (73.6%) had a tumor located in the colon and 713 (26.4%) had a tumor located in the rectum. Histologically, the frequency of mucinous adenocarcinoma (MUC) or signet-ring cell (SRC) carcinoma was 6.9% (185/2,697) in pT1 stage colorectal cancer patients. The presence of PNI was detected in 51 patients (1.9%) in pT1 stage patients. In this patient cohort, the prevalence of lymph node metastasis in pT1 stage patients was 15.2% (410/2,697), and the median of metastatic lymph nodes was 2 (IQR: 1–3). According to the pathological N category, 85.1% (349/410) of patients were classified as pN1 stage and 14.9% (61/410) of patients were classified as pN2 stage. The rectal cancer patients had a higher incidence of lymph node metastasis than colon cancer patients (18.0% vs 14.2%, *p* = 0.017). The median number of retrieved lymph nodes was 16 (IQR: 12–21), and 80.8% (2,178/2,697) of colorectal cancer patients had at least 12 lymph nodes yield.

The distribution of baseline characteristics between patients with and without lymph node metastasis were shown in Table1. The results showed that age was older (*p* < 0.001), the proportions of rectum (*p* = 0.017), elevated CEA level (*p* = 0.038) as well as poorly differentiation/MUC/SRC (*p* < 0.001) were higher, and PNI (*p* < 0.001) was more frequent in patients with lymph node metastasis than those without lymph node metastasis (Table1).

**TABLE 1:** Comparison of baseline characteristics of pT1 stage colorectal cancer patients with and without lymph node metastasis.

Characteristics	Patients (%)	Lymph node metastasis		
		No (*n* = 2,287)	Yes (*n* = 410)	*p* Value
Age (years)				<0.001
<60	1,105 (41.0%)	890 (38.9%)	215 (52.4%)	
≥60	1,592 (59.0%)	1,397 (61.1%)	195 (47.6%)	
Sex				0.873
Female	1,253 (46.5%)	1,064 (46.5%)	189 (46.1%)	
Male	1,444 (53.5%)	1,223 (53.5%)	221 (53.9%)	
Race				0.130
White	2,147 (79.6%)	1832 (80.1%)	315 (76.8%)	
Black/Other	550 (20.4%)	455 (19.9%)	95 (23.2%)	
Tumor location				**0.017**
Colon	1984 (73.6%)	1702 (74.4%)	282 (68.8%)	
Rectum	713 (26.4%)	585 (25.6%)	128 (31.2%)	
CEA level				**0.038**
Normal	2,234 (82.8%)	1909 (83.5%)	325 (79.3%)	
Elevated	463 (17.2%)	378 (16.5%)	85 (20.9%)	
Differentiation type				**<0.001**
WD/MD	2,320 (86.0%)	2001 (87.5%)	319 (77.8%)	
PD/MUC/SRC	377 (14.0%)	286 (12.5%)	91 (22.2%)	
Tumor size (cm)				0.942
≤2 cm	1,548 (57.4%)	1,312 (57.4%)	236 (57.6%)	
>2 cm	1,149 (42.6%)	975 (42.6%)	174 (42.4%)	
Perineural invasion				**<0.001**
No	2,646 (98.1%)	2,262 (98.9%)	384 (93.7%)	
Yes	51 (1.9%)	25 (1.1%)	26 (6.3%)	
Lymph node yield				0.348
<12	519 (19.2%)	447 (19.5%)	72 (17.6%)	
≥12	2,178 (80.8%)	1840 (80.5%)	338 (82.4%)	

WD, well-differentiated; MD, moderately differentiated; PD, poorly differentiated; MUC, mucinous adenocarcinoma; SRC, signet-ring cell carcinoma. The bold values were used to highlight a significantly statistical significance (*p* < 0.05).

### Univariate and Multivariate Analysis of Predictive Factors for Lymph Node Metastasis in pT1 Stage Patients

The univariate and multivariate logistic regression analysis for lymph node metastasis in pT1 stage colorectal cancer patients were summarized in Table 2. A total of five variables, including patient age (*p* < 0.001), tumor location (*p* = 0.017), differentiation type (*p* < 0.001), CEA level (*p* = 0.038) and the presence of PNI (*p* < 0.001), were shown to be associated with increased risk of lymph node metastasis according to the results of univariate analysis. After adjusting for potential covariates, independent predictive factors for lymph node metastasis were identified as patient age less than 60 years old (OR:1.869, 95% CI: 1.505–2.321, *p* < 0.001), poorly differentiated or mucinous or SRC adenocarcinoma (OR:2.075, 95% CI: 1.584–2.717, *p* < 0.001), elevated CEA level (OR:1.343, 95% CI: 1.022–1.763, *p* = 0.033) and PNI (OR:6.212, 95% CI: 3.502–11.017, *p* < 0.001) (Table 2).

**TABLE 2:** Univariate and multivariate analysis of predictive factors for lymph node metastasis in pT1 stage colorectal cancer patients.

Factors	Univariate analysis		Multivariate analysis	
	OR (95% CI)	*p* Value	OR (95% CI)	*p* Value
Age (year)		<0.001		<0.001
≥60	Reference		Reference	
<60	1.731 (1.401–2.138)		1.869 (1.505–2.321)	
Sex		0.873		
Female	Reference			
Male	1.017 (0.824–1.256)		—	
Race		0.130		
White	Reference			
Black/Other	0.801 (0.621–1.032)		—	
Tumor location		**0.017**		0.274
Colon	Reference		Reference	
Rectum	1.321 (1.050–1.660)		1.144 (0.899–1.454)	
CEA level		**0.038**		**0.033**
Normal	Reference		Reference	
Elevated	1.321 (1.015–1.718)		1.343 (1.022–1.763)	
Differentiation type		**<0.001**		**<0.001**
WD/MD	Reference		Reference	
PD/MUC/SRC	1.996 (1.533–2.598)		2.075 (1.584–2.717)	
Tumor size (cm)		0.942		
≤2 cm	Reference			
>2 cm	0.992 (0.802–1.227)		—	
Perineural invasion		**<0.001**		**<0.001**
No	Reference		Reference	
Yes	6.126 (3.501–10.720)		6.212 (3.502–11.017)	
Lymph node yield		0.348		
<12	Reference			
≥12	1.140 (0.867–1.501)		—	

WD, well-differentiated; MD, moderately differentiated; PD, poorly differentiated; MUC, mucinous adenocarcinoma; SRC, signet-ring cell carcinoma. The bold values were used to highlight a significantly statistical significance (*p* < 0.05).

### The Prognostic Significance of Lymph Node Metastasis for pT1 Stage Colorectal Cancer Patients

**FIGURE 1:**
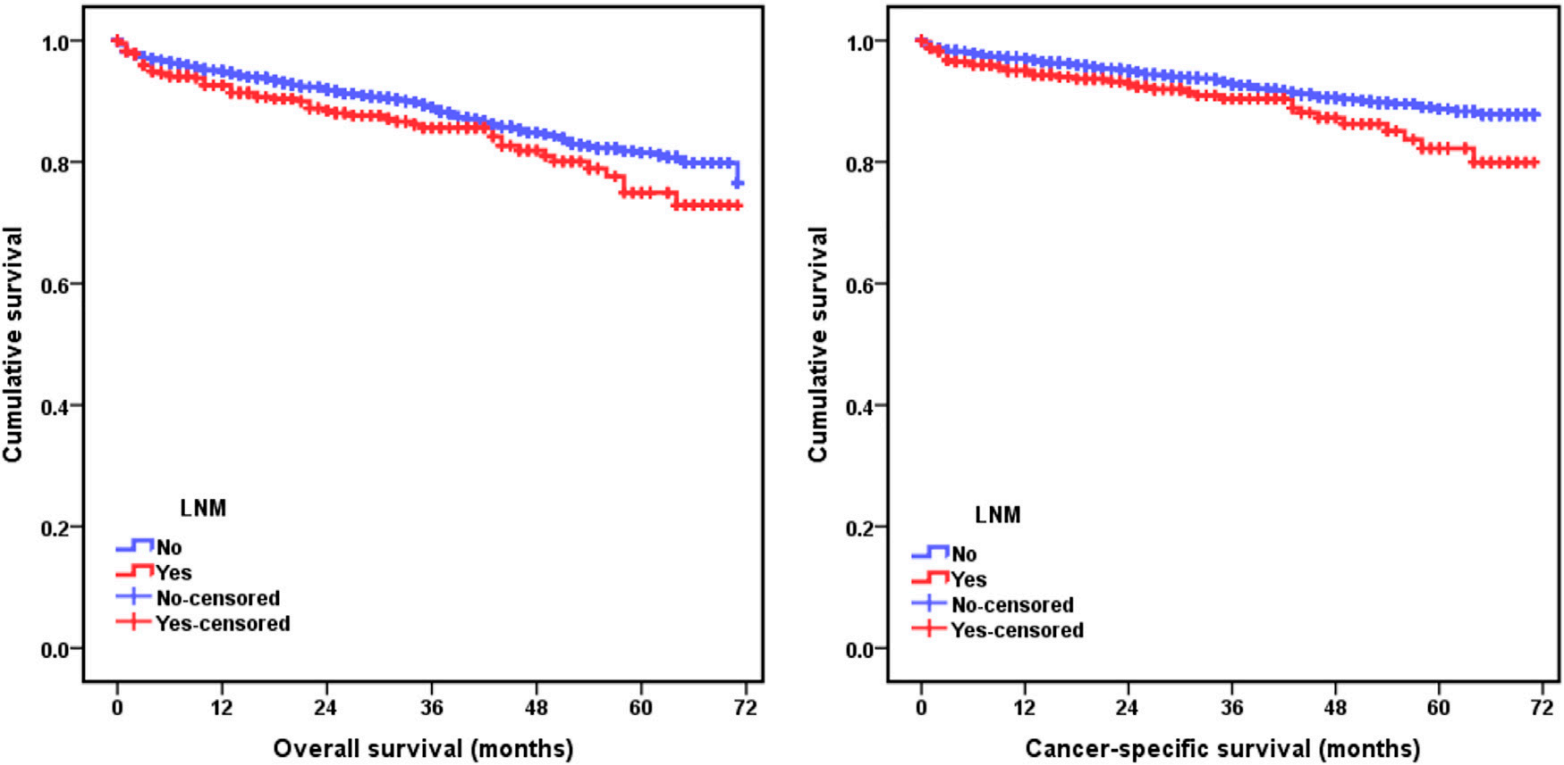
Kaplan-Meier curves for comparison of overall survival (OS) and cancer-specific survival (CSS) between pT1 stage colorectal cancer patients with and without lymph node metastasis.

The relationships between lymph node metastasis and the prognosis of pT1 stage colorectal cancer patients were further investigated by the Kaplan-Meier curves. The survival analysis demonstrated that pT1 stage patients with lymph node metastasis had a worse OS (5-year OS: 82.2% vs 88.7%, *p* = 0.020) and CSS (5-year CSS: 74.9% vs 81.5%, *p* = 0.041) than those without lymph node metastasis (Figure 1). The univariate and multivariate Cox analysis further revealed the prognostic significance of lymph node metastasis for pT1 stage colorectal cancer patients, and the analysis results demonstrated that it was an independent predictor of poor OS (HR: 1.543, 95% CI: 1.156–2.060, *p* = 0.003) and CSS (HR: 1.614, 95% CI: 1.121–2.324, *p* = 0.010) (Tables 3, 4).

**TABLE 3:** Univariate and multivariate analysis of predictive factors for OS in pT1 stage colorectal cancer patients.

Factors	Univariate analysis		Multivariate analysis	
	HR (95% CI)	*p* Value	HR (95% CI)	*p* Value
Age (year)		<0.001		<0.001
<60	Reference		Reference	
≥60	4.014 (2.966–5.433)		4.004 (2.951–5.432)	
Sex		0.112		
Female	Reference			
Male	1.201 (0.959–1.504)		—	
Race		0.821		
White	Reference			
Black/Other	1.033 (0.778–1.372)		—	
Tumor location		**0.024**		0.597
Colon	Reference		Reference	
Rectum	0.733 (0.560–0.960)		0.929 (0.707–1.220)	
CEA level		**<0.001**		**<0.001**
Normal	Reference		Reference	
Elevated	1.873 (1.458–2.405)		1.697 (1.321–2.182)	
Differentiation type		**0.007**		**0.049**
WD/MD	Reference		Reference	
PD/MUC/SRC	1.476 (1.112–1.959)		1.332 (1.001–1.772)	
Tumor size (cm)		0.268		
≤2 cm	Reference			
>2 cm	1.134 (0.908–1.418)		—	
Perineural invasion		**0.013**		0.182
No	Reference		Reference	
Yes	2.024 (1.161–3.528)		1.474 (0.834–2.605)	
Lymph node yield		0.581		
<12	Reference			
≥12	0.927 (0.710–1.212)		—	
Lymph node metastasis		**0.043**		**0.003**
No	Reference		Reference	
Yes	1.342 (1.009–1.785)		1.543 (1.156–2.060)	

WD, well-differentiated; MD, moderately differentiated; PD, poorly differentiated; MUC, mucinous adenocarcinoma; SRC, signet-ring cell carcinoma. The bold values were used to highlight a significantly statistical significance (*p* < 0.05).

**TABLE 4:** Univariate and multivariate analysis of predictive factors for CSS in pT1 stage colorectal cancer patients.

Factors	Univariate analysis		Multivariate analysis	
	HR (95% CI)	*p* Value	HR (95% CI)	*p* Value
Age (year)		<0.001		<0.001
<60	Reference		Reference	
≥60	4.244 (2.857–6.303)		4.180 (2.805–6.228)	
Sex		0.118		
Female	Reference			
Male	1.260 (0.943–1.683)		—	
Race		0.277		
White	Reference			
Black/Other	1.238 (0.842–1.819)		—	
Tumor location		0.236		
Colon	Reference			
Rectum	0.817 (0.584–1.141)		—	
CEA level		**<0.001**		**<0.001**
Normal	Reference		Reference	
Elevated	2.169 (1.590–2.958)		1.941 (1.421–2.651)	
Differentiation type		**0.003**		**0.025**
WD/MD	Reference		Reference	
PD/MUC/SRC	1.684 (1.188–2.387)		1.496 (1.053–2.126)	
Tumor size (cm)		0.606		
≤2 cm	Reference			
>2 cm	1.078 (0.810–1.436)		—	
Perineural invasion		**0.002**		**0.047**
No	Reference		Reference	
Yes	2.719 (1.438–5.141)		1.940 (1.010–3.727)	
Lymph node yield		0.420		
<12	Reference			
≥12	0.870 (0.621–1.220)		—	
Lymph node metastasis		**0.021**		**0.010**
No	Reference		Reference	
Yes	1.513 (1.064–2.152)		1.614 (1.121–2.324)	

WD, well-differentiated; MD, moderately differentiated; PD, poorly differentiated; MUC, mucinous adenocarcinoma; SRC, signet-ring cell carcinoma. The bold values were used to highlight a significantly statistical significance (*p* < 0.05).

## Discussion

Lymph node metastasis is one of the most important prognostic factors for early colorectal cancer patients [14], which determined the clinical management strategies of these patients. Submucosal invasive (pT1 stage) colorectal cancer is usually considered less advanced and has a relatively low risk of lymph node metastasis, which makes endoscopic submucosal resection possible. According to the previous reports, lymph node metastasis could occur in approximately 10.1%–17.0% of pT1 stage colorectal cancer patients [5,15–18]. Lymphovascular invasion, poorly differentiated adenocarcinoma or mucinous or signet-ring cell carcinoma, deep submucosal invasion (≥1,000 μm) and tumor budding have been reported to be associated with increased risk of lymph node metastasis in pT1 stage colorectal cancer patients [5–8,19]. The current treatment guidelines recommended that patients with these high-risk factors should receive additional surgical treatment with lymph node dissection [9,10]. However, only 6.8%–14.0% of submucosal invasive colorectal cancer patients were pathologically confirmed as the presence of lymph node involvement after additional surgical resection [12,13]. Therefore, accurate identification of predictive factors for lymph node metastasis in pT1 stage colorectal cancer patients is absolutely necessary to determine who would be eligible for endoscopic resection.

In the present study, our results demonstrated that lymph node metastasis was an independent prognostic factor for pT1 stage colorectal cancer patients. Patients aged less than 60 years old, poorly differentiated or mucinous or SRC adenocarcinoma, elevated CEA level and PNI were identified as independent predictive factors for lymph node metastasis. It is well established that the presence of lymph node metastasis could be well predicted by lymphovascular invasion (LVI). Unlike the presence of LVI, the potential impact of PNI on lymph node metastasis for pT1 stage colorectal cancer patients may be underestimated. Although previous reports have been shown that PNI was an independent predictive factor for poor survival in colorectal cancer patients [20,21], the clinical significance of PNI for pT1 stage patients did not receive a high research attention. In this series, the incidence of PNI was only 1.9% in pT1 stage patients, but the risk of lymph node metastasis increased 6.2 fold for patients with the presence of PNI in comparison to those without PNI. Similarly, Huh et al revealed PNI was a useful indicator to predict lymph node metastasis in early colorectal cancer patients, with a OR value of 10.745 [22]. The presence of PNI was considered as an important pathway for the local spread and distant metastasis of tumor cells, and it may represent an aggressive biological behavior of the tumor [23]. We believed that routine pathological assessment for the presence of PNI should be considered for determining the risk of lymph node metastasis in pT1 stage colorectal cancer patients.

Another important finding of the current study was the potential predictive role of elevated CEA level for lymph node metastasis. Recent studies have shown a significant association between elevated CEA level and tumor recurrence or poor survival in colorectal cancer patients [24,25]. However, the clinical significance of serum tumor biomarkers has not been established fully in early colorectal cancer patients. In a previous study, Sun et al reported that preoperative CEA, CA199 and CA724 level were associated with lymph node metastasis in pT1 stage colorectal cancer patients, but only elevated CA724 level was an independent risk factor for lymph node metastasis [14]. In the present study, 17.2% of pT1 stage colorectal cancer patients had an elevated CEA level before operation. Increased expression and release of preoperative serum CEA may reveal a relatively high tumor burden and biological aggressiveness. Our results suggested that CEA level may provide valuable information for risk assessment of lymph node metastasis in pT1 stage colorectal cancer patients. If so, the serum biomarker test for CEA would be expected to further improve the prediction accuracy.

In clinical practice, endoscopic resection seems to be more feasible for rectal cancer compared with colon cancer patients. However, previous studies have reported that rectal cancer patients had a higher risk of lymph node metastasis and a larger proportion of LVI than colon cancer patients [6,26]. Anatomically, the rectum is an organ with rich blood supply and has well-developed collateral circulation. Theoretically, its lymphatic and capillary vessel may be more likely to be invaded by tumor. In this study, we found that the frequency of lymph node metastasis was higher in pT1 stage rectal cancer patients than in pT1 stage colon cancer patients (18.0% vs 14.2%, *p* = 0.017). However, tumor location failed to show a significant correlation with lymph node metastasis based on the multivariate logistic regression analysis. Similar findings have been reported in other studies, lymph node metastasis for pT1 stage colorectal cancer patients may be not associated with a specific site in the colon or rectum [27,28]. In addition, patient age was identified as a risk factor for lymph node metastasis in this study. Patient-related factors such as age and comorbidity were important considerations when deciding whether or not to perform a surgical intervention for pT1 stage colorectal cancer. Fortunately, younger patients were more likely to had a relatively low risk of postoperative morbidity and mortality compared with their older counterparts. In terms of benefit-risk balance, young pT1 stage patients with one or more risk factors may be the optimum candidates for surgical intervention.

It has been demonstrated that no single clinicopathologic feature of colorectal cancer could accurately predict the risk of lymph node metastasis. Recently, a few nomogram models were developed to provide risk stratification of lymph node metastasis for pT1 stage colorectal cancer patients. Using data from the training cohort and an external validation cohort, Oh et al proposed a nomogram model containing vascular invasion, differentiation type, the depth of submucosal invasion, tumor budding and background adenoma to predict the risk of lymph node metastasis in pT1 stage patients [8]. The results showed a C-index of 0.812 (95%CI: 0.770–0.855) for the predictions of lymph node metastasis in the training cohort, and the predictive performance was still excellent for the external validation cohort, with a C-index of 0.771 (95%CI: 0.708–0.834) [8]. This quantified prediction model showed a good discrimination and conformance for the risk prediction of lymph node metastasis. In our study, however, we did not establish a predictive model based on the identified clinicopathologic variables due to the lack of key histopathological parameters. We expected that the data from the current study could further improve predictive performance of the nomogram model for lymph node metastasis in pT1 stage colorectal cancer patients.

Several limitations of this study require further discussion. Firstly, only those patients with complete data were included and analyzed, which could introduce the potential selection bias. Secondly, the relevant information on PNI was not systematically collected in the SEER database before 2010, thus previous cases were not enrolled in the study cohort. Thirdly, several well-established histopathological parameters such as lymphovascular invasion, the depth of submucosal invasion and tumor budding were not included in this analysis because they were unavailable in the SEER database. This made the current analysis miss some important pathological information. It is possible that risk factors identified by the SEER dataset would be no more significant if histopathological parameters such as lymphovascular invasion, the depth of submucosal invasion and tumor budding were concurrently included in the multivariate regression analysis. A nomogram model that integrates factors identified by the current analysis with these pathological variables needs to be constructed to test its clinical validity, but incomplete data from the SEER database does not allow us to do it.

Taken together, based on a real world population-based analysis, our results demonstrated that patients aged less than 60 years old, poorly differentiated or mucinous or SRC adenocarcinoma, elevated CEA level and PNI were independent predictive factors for lymph node metastasis in pT1 stage patients. These findings might help clinicians identify high-risk individuals with lymph node metastasis.

## Data Availability

The raw data supporting the conclusions of this article will be made available by the authors, without undue reservation.
